# Molecular classification of soft tissue sarcomas for adequate diagnosis: A study on the northeast population of Morocco

**DOI:** 10.1016/j.heliyon.2022.e10673

**Published:** 2022-09-17

**Authors:** Rhizlane El Koubaiti, Asmae Mazti, Mustapha Maaroufi, Mohammed EL Idrissi, Abdelhalim El Ibrahimi, Abdelmajid El Mrini, Touria Bouhafa, Samira El Fakir, Karim Ouldim, Samia Arifi, Laila Chbani

**Affiliations:** a Biomedical and Translational Research Laboratory, Faculty of Medicine and Pharmacy, Sidi Mohamed Ben Abdellah University, Fez, Morocco; b Pathology Department. Hassan II University Hospital, Faculty of Medicine and Pharmacy Fez, Sidi Mohamed Ben Abdellah University, Fez, Morocco; c Department of Radiology, Hassan II University Hospital, Faculty of Medicine and Pharmacy Fez, Sidi Mohamed Ben Abdellah University, Fez, Morocco; d Department of Trauma-orthopedics B4, Hassan II University Hospital, Faculty of Medicine and Pharmacy Fez, Sidi Mohamed Ben Abdellah University, Fez, Morocco; e Department of Radiotherapy, Hassan II University Hospital, Faculty of Medicine and Pharmacy Fez, Sidi Mohamed Ben Abdellah University, Fez, Morocco; f Department of Epidemiology and Public Health, Hassan II University Hospital, Faculty of Medicine and Pharmacy Fez, Sidi Mohamed Ben Abdellah University, Fez, Morocco; g Laboratory of Medical Genetics and Oncogenetics, HASSAN II University Hospital, Fez, Morocco; h Medical Oncology Department, Faculty of Medicine and Pharmacy Fez, Sidi Mohamed Ben Abdellah University, Fez, Morocco

**Keywords:** Soft tissue sarcomas, Diagnosis, Fluorescence in situ Hybridization, Molecular classification

## Abstract

**Background:**

Soft tissue sarcomas (STS) are a heterogeneous group of tumors. For adequate therapeutic management, an accurate diagnosis is necessary. In Morocco, the diagnosis is essentially based on the morphological and immunohistochemical study. Compared to other techniques, fluorescence in situ hybridization (FISH) is easier to develop and less expensive. This study aims to assess the feasibility and utility of implementing FISH technique to improve diagnostic accuracy and establish a good classification.

**Material and methods:**

This is a retrospective cohort study. 211 cases of mesenchymal tumors were included. Hematoxylin Eosin Safran (HES) staining was performed in all cases followed by immunohistochemistry (IHC). FISH was performed in all cases with suspected STS. The probes used were EWSR1, MDM2 and SS18. The performance of FISH and histopathological test were evaluated by the ROC curve method (receiver operating characteristic). We evaluated the concordance between FISH and real time PCR by Cohen test.

**Results:**

The real-time PCR technique showed good agreement with the FISH test by a Kappa coefficient of 60% (p = 0.035). FISH was able to confirm that it is more accurate (Youden’s Index = 91%) than histological/immunohistochemical analysis (Youden’s Index = 51%), as well as the positive predictive value was higher (100%) with an ROC curve finding a larger area under the curve of 0.953 (95% CI: 0.918–0.988), p = 0.000 which supports that FISH shows high performance to present an accurate final diagnosis.

**Conclusion:**

This is the first and the largest Moroccan series for the molecular diagnosis of STS by FISH. Our study shows that paraffin FISH is a sensitive and specific ancillary tool in the diagnosis of STS when used in the appropriate clinicopathological context.

## Highlights

• Access to molecular diagnosis for STS patients has been a challenge in Morocco, resulting in delayed or inaccurate diagnosis.
• FISH is an essential and affordable technique that can be implemented with a relative ease for the diagnosis of soft tissue sarcomas.
• Increased communication and networking may help to make substantial improvement in sarcoma diagnosis in Morocco.

## 1 Introduction

Soft tissue sarcomas (STS) are a heterogeneous group of solid malignancies, accounting for less than 1% of all malignant tumors in adults [1]. More than 100 histological subtypes have been described in the recent WHO classification with distinct clinical behaviors and aggressivenesses [2]. These tumors can affect patients at any age but they have a high prevalence in patients over 40 years old [3]. They can occur in any part of the body. Their rarity and the absence of specific morphological features or specific immunohistochemical markers make them real challenges for the pathologists [4].

Fluorescent In Situ Hybridization (FISH) is one of the most reliable and practical genetic approaches for the diagnosis of sarcoma cases, especially those with a simple karyotype, including sarcomas with specific translocations or amplifications [5, 6, 7].

The implementation of molecular testing for STS in an economically-constrained context is very challenging. However, we believe that FISH represents a minimum requirement for a pathology department treating sarcomas cases and must be implemented as a routine method.

In this paper we report our experience as the first local pathology department providing FISH testing for the diagnosis of sarcoma patients in Morocco.

The purposes of this study were to assess the feasibility and utility of implementing the FISH technique in a local laboratory, to provide the most accurate epidemiology based on molecular diagnosis for the first time, and to suggest an adapted algorithm for molecular testing in the context of limited resources based on morphology and IHC pre-screening.

## 2 Materials and methods

This was a retrospective cohort study that was completed between September 2010 and September 2021 involving 211 cases of soft tissue tumors classified based on cellular type: 74 cases with round cells, 59 cases with spindle cells, 69 cases of atypical adipose tumors, and 9 cases with pleomorphic cells.

**Figure 1:**
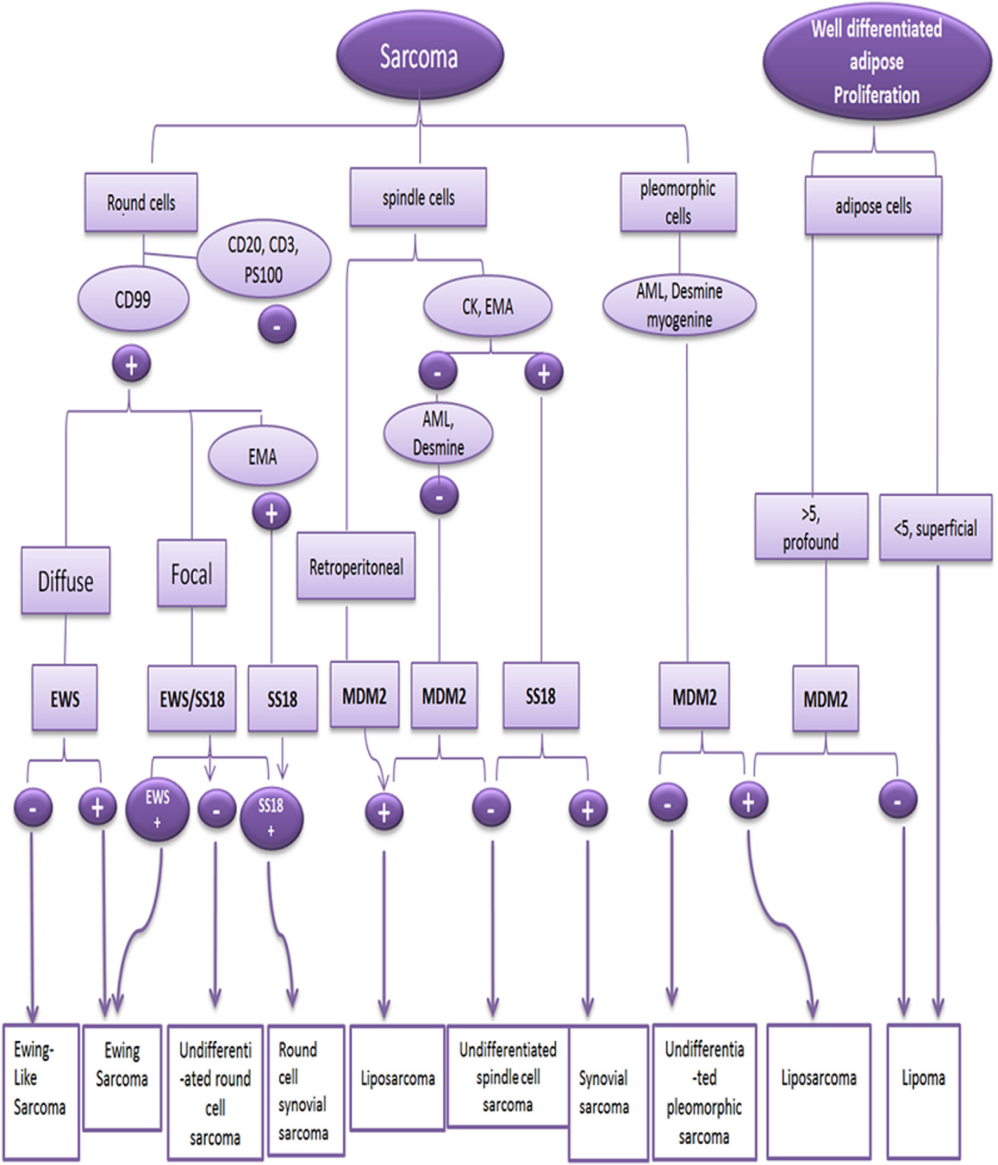
The steps followed in our study for the diagnosis of soft tissue sarcomas.

All selected patients were from the northeast region of Morocco and were diagnosed in the Department of Pathology of the Hassan II Teaching Hospital of Fez. Clinical and histopathological data were recorded from pathology application forms and patient medical records. The steps for our study of STS diagnosis are illustrated in Figure 1.

The most sought-after genetic aberrations in our department are the fusion of the EWSR1 gene for the Ewing sarcoma family (EWS), the t(X; 18) translocation for synovialosarcoma (SS), FOXO1 in the case of alveolar rhabdomyosarcoma (ARMS), and the amplification of the MDM2 gene for atypical lipomatous tumors (ALT)/well-differentiated liposarcoma (WDLS) and differentiated liposarcomas (DDLS).

### 2.1 Histological diagnosis

Hematoxylin-eosin saffron (HES) analysis was performed for all cases. Sections of 5 μm thickness were prepared from FFPE tissue blocks and stained with HES.

### 2.2 Immunohistochemical diagnosis

Specific antibodies for each type of tumor were used according to the manufacturer’s instructions with an automated immunohistochemical dye (Ventana BenchMark ULTRA®). Immunohistochemistry was performed using anti-CD99, anti-Cytokeratin (CK), Cytokeratin (CK) anti epithelial membrane antigen (anti-EMA), anti-smooth muscle actin (anti-AML), anti-desmin, H-caldesmon (H-CD), CD20, and CD3, and PS100 was used to eliminate other histological subtypes.

### 2.3 Molecular diagnosis

Ambiguous cases were selected for FISH assessment. Paraffin sections 3.5 μm thick were mounted on positively charged slides (Super Frost). Then they were deparaffinized at 60 °C overnight. The slides were treated with a 1X SSC hot buffer wash at 80 °C (35 min) then with a proteolytic enzymatic treatment at 37 °C, and finally they were washed with distilled water and dehydrated with a series of alcohols. The tissues were subjected to FISH analysis according to the instructions mentioned in the literature for the FISH probe. The FISH probes used were Vysis FISH, Abbott Laboratories® EWSR1, 22q12 double-break Break Apart Rearrangement Probe for Ewing’s sarcoma, the Spectrum Orange Vysis LSI MDM2 FISH probe targeted to the 12q15 region on chromosome 12 for liposarcoma, and the Vysis LSI SS18 (18q11.2) Bi-Color Rearrangement Probe for synovial sarcomas. The slides were placed in ThermoBrite® on a co-denaturation program at 73 °C for 5 min then hybridization at 37 °C for 20 h. After hybridization the slides were washed with the washing buffers NP40 0.3% and 0.1% and FISH slides were analyzed on a Leica DM 2500 epifluorescence microscope using a DAPI/Green/Red triple-band filter at a magnification of 100X. The threshold of positivity for the Break Apart Probe was a 30% nucleus with 3 spots and 6 MDM2 copies. In the case of translocated sarcomas, for each sample a minimum of 100 non-overlapping tumor cells were evaluated to assess the presence of fused or split green and red signals. A positive result was defined as >30% of cells with split signals.

Using the TaqMan® Fast Universal PCR Master Mix (2X) No Amperase® UNG kit, we were able to search for the fusion of the SSX-SYT gene by RT-PCR for ten patients suspected of having synovial sarcoma. A highly positive and a negative control were used to monitor the amplification stages of fusion genes and endogen (B2M). Probes and primers used are listed in Table 1.

**Table 1:** Primer and probe sequences used in real time PCR testing.

Primer and probe name	Primer and Probe
SSX-C R	5′-CRT TTT GTG GGC CAG ATG C- 3′
SYT-B F	5′-AGA GGC CTT ATG GAT ATG ACC AGA T-3′
SSX1	FAM - TCC CTT CGA ATC ATT TTC GTC CTC TGC T - TAMRA
SSX2	FAM - TCT GGC ACT TCC TCC GAA TCA TTT CCT T - TAMRA

### 2.4 Survival analysis

The patients were subdivided into two groups. Group 1 included patients treated before FISH test results and Group 2 included patients treated after FISH test results. Overall survival was defined as the time from the start of diagnosis until death or until the last follow-up. Relapse-free survival and metastasis-free survival were measured from the date of initial diagnosis until the date of relapse, regional metastasis, or last follow-up/death.

### 2.5 Statistical analysis

Statistical analyses were conducted using the statistical software IBM SPSS 19.Ink. The Kaplan-Meier method [8] was adopted for the survival analysis and survival curves were compared with the log-rank test [9].

We used the Cohen test to analyze the concordance between different diagnostic tests.

We then evaluated the diagnostic performance of the histological and FISH tests, which was expressed in terms of sensitivity, specificity, positive predictive value (PPV), and negative predictive value (NPV) with reference to the diagnosis retained.

The different values were calculated using the following formulas [10]:

• **Sensitivity** = True Positive/(True Positive + False Negative)
• Specificity = True Negative/(True Negative + False Positive)
• PPV = True Positive/(True Positive + False Positive)
• NPV = True Negative/(True Negative + False Negative)
• Accuracy = (True Positive + True Negative)/(True Positive + True Negative + False Positive + False Negative)
• Youden Index = (Sensitivity + Specificity − 1)

The diagnostic values of the two tests were evaluated by the ROC curve method (receiver operating characteristic). For all tests, a p value less than 0.05 was considered the significance level.

### 2.6 Ethic statement

This study was approved by the ethical medical committee of the Hassan II University Hospital of Fez. Ethical Permit No. 34/16. All subjects gave written informed consent.

## 2.7 Funding

This work was supported and funded by the Institute for Research on Cancer (IRC), 10.13039/501100016992Hassan II University Hospital of Fez Morocco (grant numbers 201881/AAP2016).

## 3 Results

### 3.1 Patient characteristics

The mean age of patients was 41 ± 21 years old. The patient's ages ranged from 1 to 87 years old. There was a male predominance with a male/female ratio of 1.11 (111 males and 100 females) **(**Table2**).** A total of 226 FISH tests were performed in 211 patients; 69% of the cases were biopsies.

**Table 2:** Clinicopathologic and molecular characteristics of patients suspected of STS.

Criteria	Total N = 211
Gender	
Male	111 (52,7%)
Women	100 (47,3%)
Age	
Median (min-max)	40 (1–87 years)
<20	50 (23,7%)
21–40	57 ​(27%)
41–60	61 (29%)
61–80	39 (18,3)
>80	4 (2%)
Tumor size (cm)	
Mean	7,8 (0,1–82 cm)
Median	5
Localization	
extremity	155 (73,4%)
Trunk	31 (14,6%)
head and neck	10 (4,7%)
Retroperitoneal	7 (3,3%)
Viscera	8 (4%)
Cell morphology	
Adipocyte proliferation	69 (32,7%)
spindle cell proliferation	59 (28%)
Small round cell proliferation	70 (33%)
Pleomorphic cell proliferation	9 (4,3%)
Others	4 (2%)
FISH test	
Interpretable	201 (95,2%)
Not interpretable	10 (4,8%)
Probes used	
EWSR1	63 (29 %)
SS18	40 (19 %)
MDM2	96 (44,5 %)
FOXO1	1 (0,5 %)
EWSR1/SS18	11 (5 %)
MDM2/SS18	4 (2 %)
Metastasis	
M0	99 (47%)
M1	32 (15%)
MX	80
Recidivism	
R0	108 (51%)
R1	23 (11%)
RX	80

MX, RX: missing data

### 3.2 Survival outcomes

**Figure 2:**
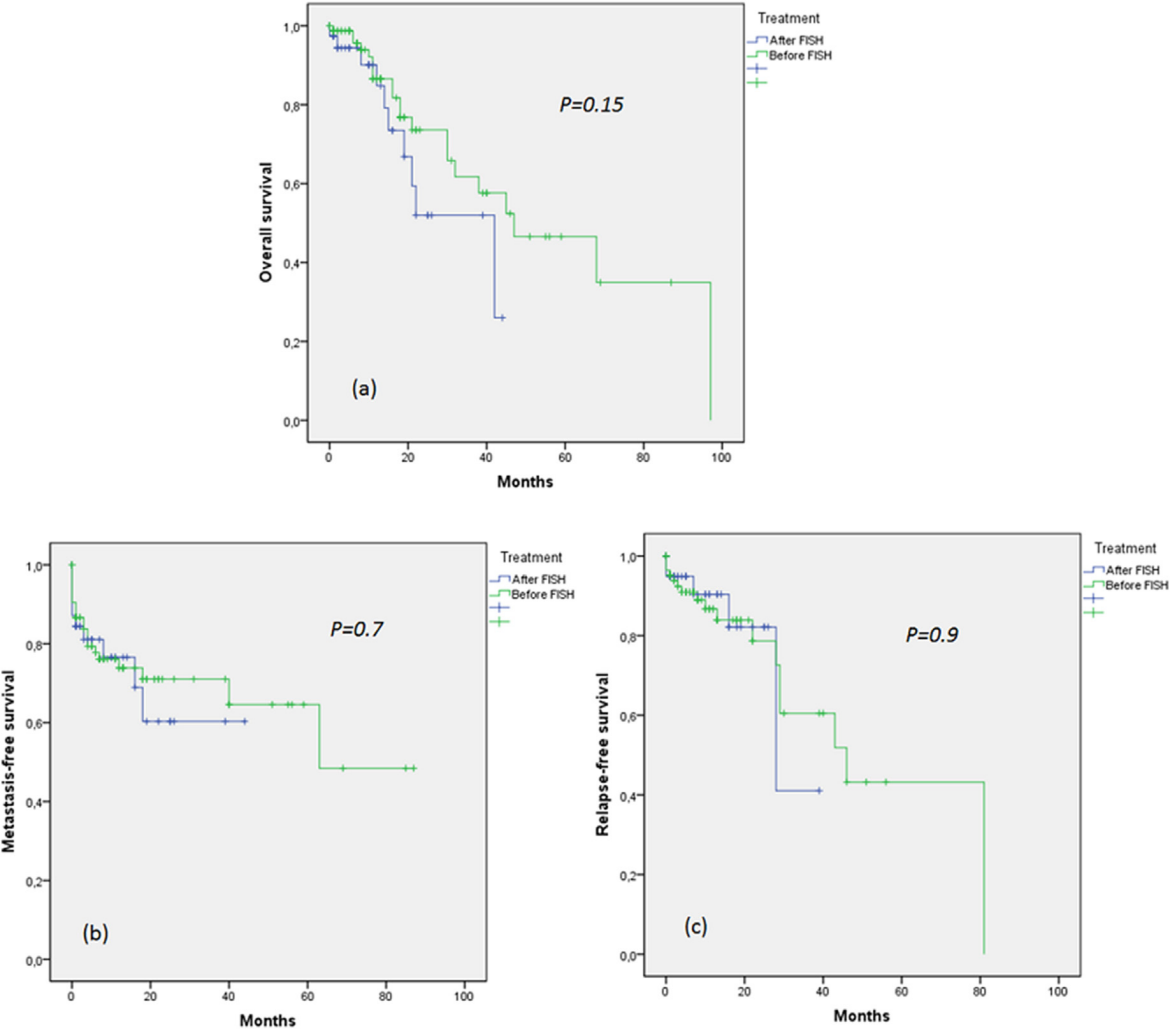
Patient survival of studied groups ((a) overall survival, (b) metastasis-free survival, and (c) relapse-free survival).

As shown in Figure 2, during the course of the study, 37 patients died. The median duration of overall survival was 45 months (range 0–97). Of 211 patients with suspected STS, 32 patients (15%) developed metastases during the follow-up period. Time to metastasis ranged from 0 to 87 with a median of 63 months. Regarding recurrence, 23 of 211 patients developed disease relapse. The time to relapse ranged from 0 to 81 months (median = 46 months).

There was no significant overall survival (p = 0.15) (Figure 2a), metastasis-free survival (p = 0.7) (Figure 2b), and relapse-free survival (p = 0.9) (Figure 2c) differences between patient treated after FISH and patients treated before FISH.

### 3.3 Classification by histological and cytogenetic study

**Figure 3:**
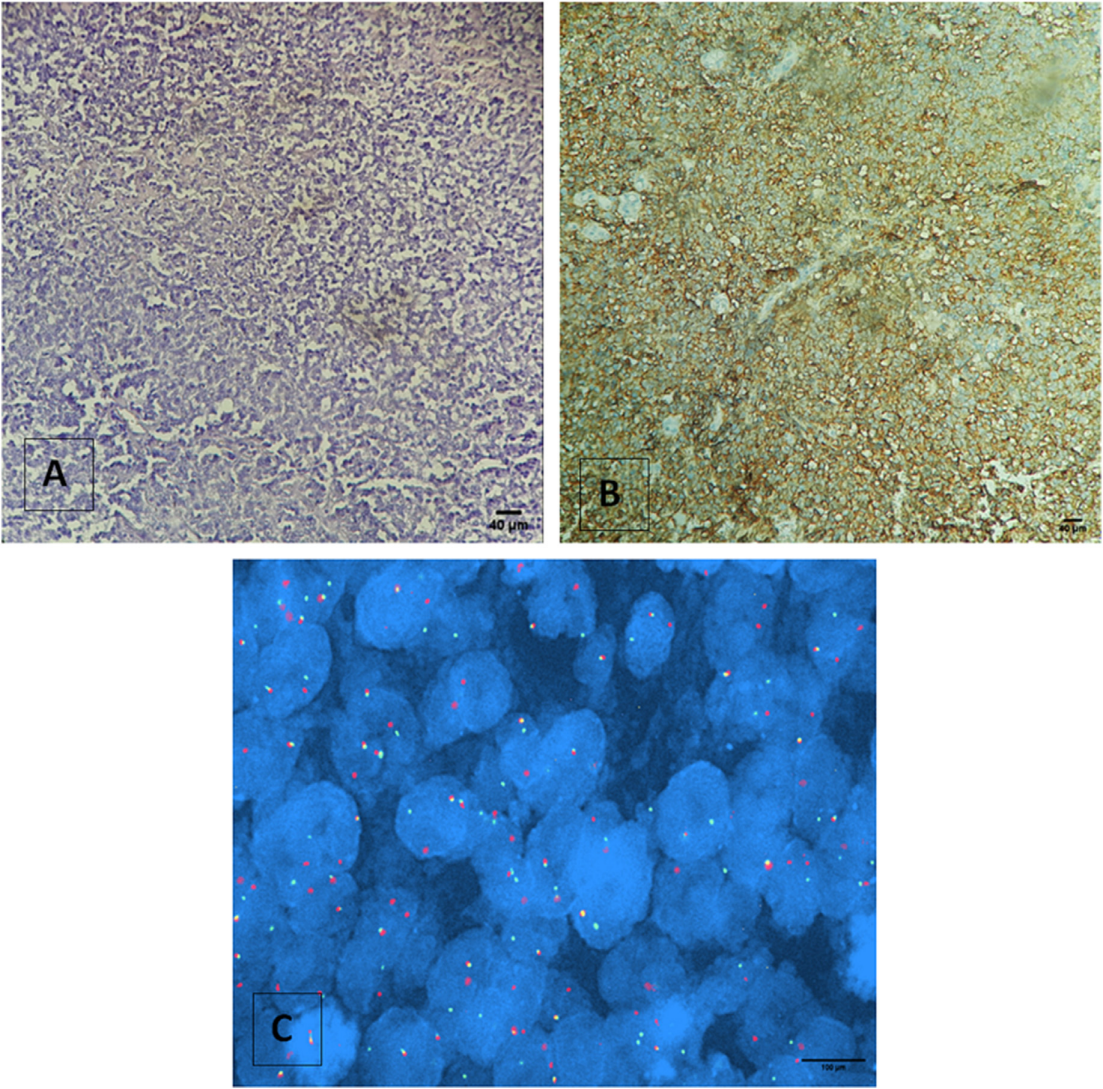
Light Microscopic Appearance of Ewing’s Sarcoma: A. Histological picture of round-cell sarcoma (Scale bar = 40 μm) B. positive immunohistochemical staining for CD99 (Scale bar = 40 μm) C. FISH technique showing break apart signal representing EWSR1 gene rearrangement (Scale bar = 100 μm).

Of the 74 suspected cases of Ewing’s sarcoma, 63 cases were diagnosed as Ewing’s sarcoma (based on histology and immunohistochemistry results), of which 28 were females and 35 were male patients. The age range was between 1 and 74 years old with a median age of 19 years. Morphologically, 95% of diagnostic cases for Ewing's sarcomas showed a proliferation of small round cells (Figure 3A). After immunohistochemistry, 84% of cases showed an intense positivity of anti-CD99 antibodies (Figure 3B). The rearrangement of the EWSR1 gene by FISH was noted in 35 cases (56 %) (Figure 3C).

We also diagnosed other types of sarcomas for three patients with rearrangement of the EWSR1 gene: one extraskeletal myxoid chondrosarcoma, one myoepithelial carcinoma, and one desmoplastic round cell tumor. For the first case, the microscopic description showed small cells with a chondroid background. An immunohistochemical study was performed and the tumor cells weakly expressed EMA and did not express CK. These results made the histological appearance compatible with extra-skeletal myxoid chondrosarcoma. The FISH study, which was conducted to look for the rearrangement of the EWS gene, was positive. The cytogenetic aspect was compatible with the histological diagnosis of extra-skeletal myxoid chondrosarcoma. On the morphological level, the second case had small round cell proliferation with expression of cytokeratin and EMA and negative expression for anti-CD99 and a rearranged EWSR1 gene. The final diagnosis was myoepithelial carcinoma. The third case had morphologically small round cell proliferation with expression of cytokeratin and desmin markers and the EWSR1 gene rearranged.

Twenty-nine cases showed no rearrangements for the EWS gene, including 21 cases that were labeled as undifferentiated round-cell sarcoma because they did not match any other category, even after applying a large panel of immunohistochemical stains. Four cases were classified as Ewing-like group round-cell sarcoma and three cases as undifferentiated spindle cell sarcoma. The FISH result was non-interpretable in six cases because the fragments were exiguous, which did not allow us to realize a correct interpretation.

**Figure 4:**
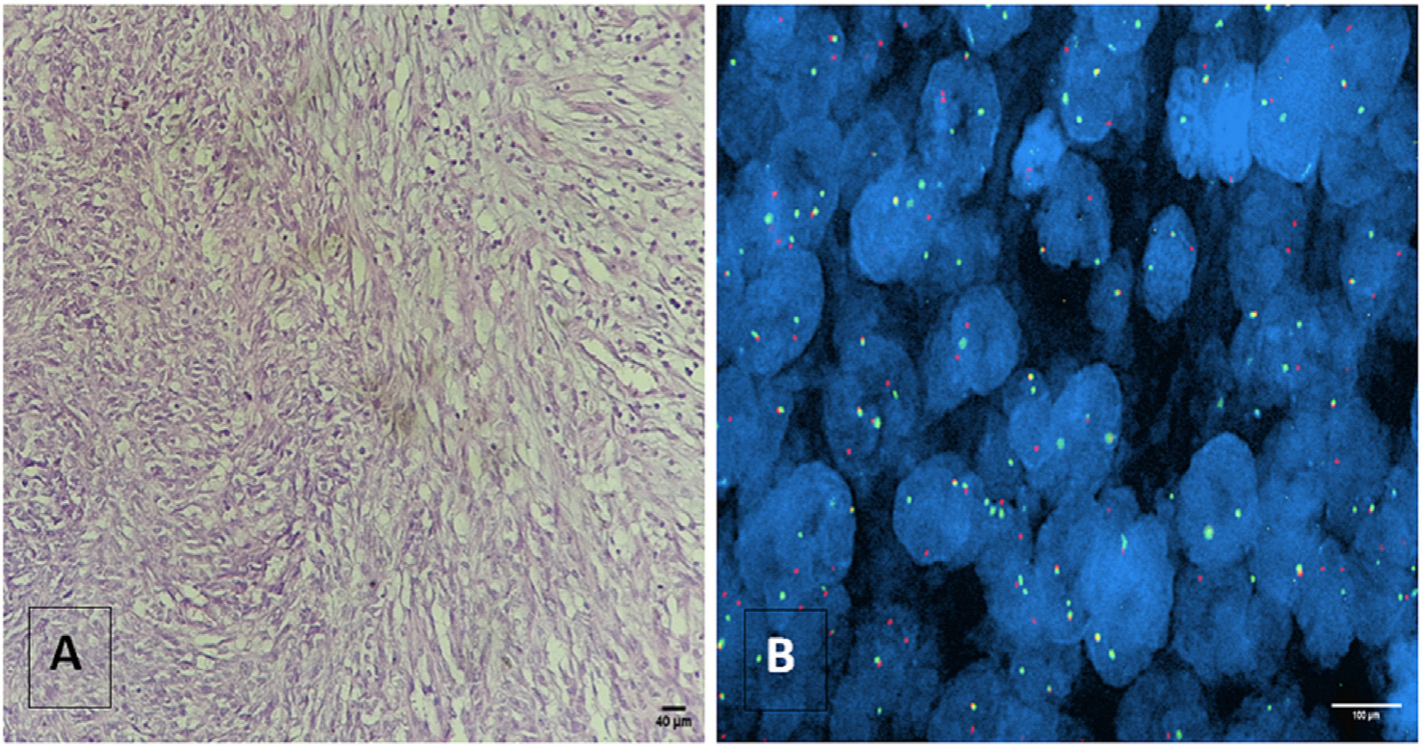
Light Microscopic Appearance of Synovial Sarcoma: A. Histological picture of spindle cells sarcoma (Scale bar = 40 μm). B. FISH technique showing break-apart signal representing SS18 gene rearrangement (Scale bar = 100 μm).

For 55 cases of suspected synovial sarcoma, 37 cases were diagnosed as SS using histology and immunohistochemistry tests. There were 15 females and 22 male patients. The ages ranged from 4 to 74 years with a median age of 34 years; 66.6% of cases showed focal positivity for CK, EMA, and CD99. The histological study showed spindle cells in 97% of the cases (Figure 4A) and small round cells in one patient who was diagnosed with cervical neuroblastoma after a negative FISH. Using the FISH technique, the 24 patients with rearranged SS18 were classified as SS (Figure 4B), while the 12 cases with non-rearranged SS18 were reported as undifferentiated sarcomas. The use of Bouin as a tissue fixative prevented the interpretation of the results in one case.

In 18 cases with inconclusive histological and immunohistochemical results, cytogenetic study by FISH confirmed the diagnosis of SS in only two cases; however, 16 cases were reclassified as undifferentiated sarcomas. The use of Bouin as a tissue fixative prevented the interpretation of the results in one case.

A total of 96 patients were suspected to have adipose tumors. The age range was 19–87 years with a median age of 54 years old.

The histopathological and immunohistochimical study detected 14 DDLS, 23 WDLS, 4 myxoid liposarcomas (MLS), 7 pleomorphic sarcomas, 3 undifferentiated sarcomas, 42 lipomas, 1 hibernoma, 1 pleomorphic leiomyosarcoma, and 1 pleomorphic rhabdomyosarcoma.

**Figure 5:**
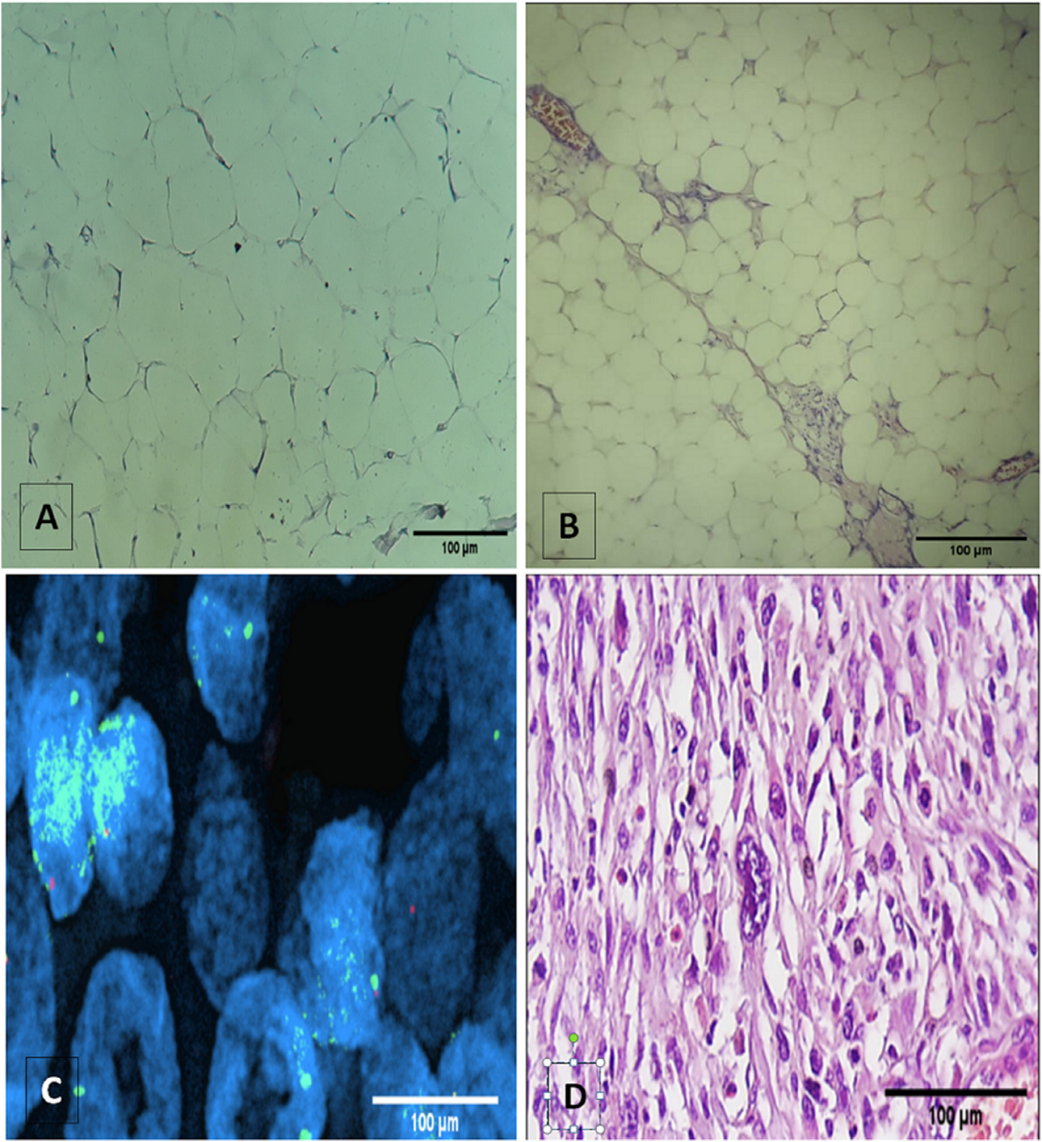
A. Light Microscopic Appearance of lipoma (Scale bar = 100 μm)B. Light Microscopic Appearance of well-differentiated Liposarcoma (Scale bar = 100 μm). C. MDM2 amplification by FISH. D. Histological picture of pleomorphic cell (Scale bar = 100 μm).

For benign lipomatous tumors, almost all cases showed adipocyte proliferation (Figure 5A) with an absence of amplification of the MDM2 gene, except for three cases with amplified MDM2 that were reclassified as WDLS **(**Table 3**)**.

**Table 3:** FISH analysis of MDM2 amplification in cases of ALT/WDLS and benign lipomatous tumors.

	FISH analysis of MDM2 amplification	
	Positive	Negative
Malignant	6	15
Benign	3	39

In our study, of the 23 cases diagnosed histologically as WDLS (Figure 5B), 15 cases were negative for MDM2 amplification and were reclassified as either undifferentiated sarcomas or lipomas. The other cases showed an amplification of the MDM2 gene (Figure 5C), except for three cases where the FISH was non-interpretable. However, 50% of DDLSs were reclassified as undifferentiated pleomorphic sarcomas after negative FISH test.

Seven cases were diagnosed with pleomorphic sarcoma. There were three males and four females. The median age was 44, ranging from 40 to 82 years old. In most cases, the histology showed pleomorphic cells (Figure 5D) and sometimes fusocellular proliferation.

A search for the amplification of the MDM2 gene using the FISH technique was carried out for all cases. Amplification was detected in three patients and the final diagnosis was in favor of a dedifferentiated liposarcoma.

The diagnosis of alveolar rhabdomyosarcoma is confirmed by looking for the FOXO1 rearrangement for a single patient and the result was in favor of the histological diagnosis.

### 3.4 Evaluation of the performance of FISH compared to histological/immunohistochemical tests

Among 135 cases that showed positivity for histological tests by referring to HES and IHC results, 80 cases showed positive results for FISH while 55 cases were negative and reclassified according to clinicopathologic, histological, and molecular data.

The final classification revealed 10 lipomas, 1 fibrosis, 33 undifferentiated sarcomas, 8 undifferentiated pleomorphique sarcomas, and 3 Ewing-like sarcomas.

For the 66 cases whose histological diagnosis was negative, the FISH test was able to detect six positive cases that escaped histological immunohistochimical diagnosis, including three pleomorphic sarcomas reclassified after FISH as dedifferentiated liposarcomas and three lipomas reclassified as well-differentiated liposarcomas.

**Figure 6:**
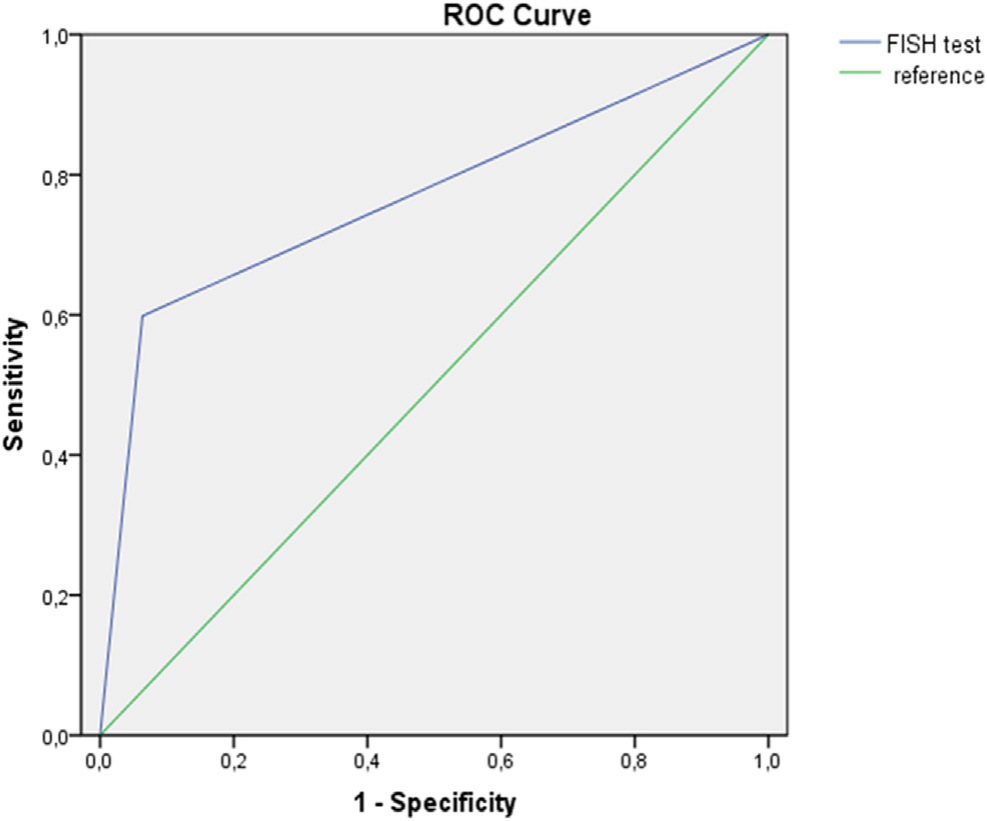
ROC curve of the FISH test with reference to IHC test.

The evaluation of the performance of the FISH test compared to the histological test was able to show a sensitivity of 59% and a specificity of 91%. However, the positive predictive value was very high with a rate of 93% **(**Table 4**)** and an ROC curve with a wide area under the curve of 0.760 (95% CI: 0.69–0.82), p = 0.000. FISH also exhibited an accuracy of 70% (Figure 6).

**Table 4:** Performance of the FISH test compared to the histological test.

	Histo/IHC+	HISTO/IHC-	Total	%
FISH+	80	6	86	PPV = 93%
FISH-	55	60	115	NPV =52%
Total	135	66	201	Accuracy = 70%
%	Sensitivity = 59%	Specificity = 91%		

PPV: positive predictive value; NPV: negative predictive value.

### 3.5 Evaluation of the performance of IHC and FISH tests compared to the final diagnosis

We evaluated the performance of histological/immunohistochimical tests and FISH in relation to the final diagnosis which was based on clinicopathological, histological, and molecular data.

The results of our study showed sensitivity (94%) of the histological tests compared to FISH, which presents only 91% **(**Table 5**)**; on the other hand, the specificity of FISH was higher (100%) than the histological tests (57%).

**Table 5:** Comparison between Histopathological and FISH test for the diagnosis of STS.

	Histological test	FISH test
Number of cases	201	201
Sensitivity	94%	91%
Specificity	57%	100%
PPV	66%	100%
NPV	91%	92%
Youden’s Index	0,51	**0,91**

PPV: positive predictive value; NPV: negative predictive value.

**Figure 7:**
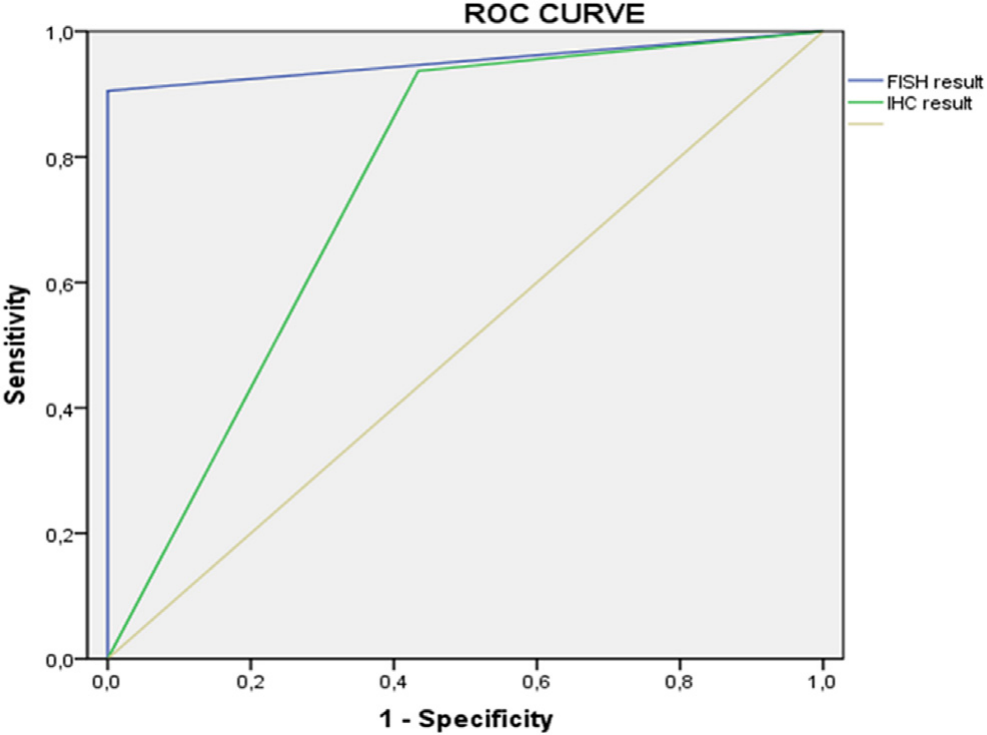
ROC curve of the IHC and FISH test with reference to the final diagnosis.

In terms of positive predictive value, the FISH technique (100%) predicts the disease better than histology (66%). The ROC curve of FISH had a larger area under the curve of 0.953 (95% CI: 0.918–0.988), p = 0.000. However, the histological test showed an ROC curve with a more or less large area under the curve of 0.751 (95% CI: 0.683–0.820), p = 0.000 (Figure 7). The FISH test’s Youden’s index (0.91) was close to the value of 1, which indicates that the FISH technique is a more accurate diagnostic tool for STS more than histological test.

### 3.6 Diagnosis by real time PCR (RT-PCR)

We performed a real-time PCR analysis in ten patients including five men and five women suspected of having synovialosarcoma. The median age was 41 years with extremes of 15–65 years old.

Nine cases showed a rearrangement of the SS18 gene in FISH. However, only eight patients were confirmed by RT-PCR. The Kappa coefficient for FISH/RT-PCR concordance was of 61.5% (p = 0.035) **(**Table 6**).**

**Table 6:** Concordance between FISH test and real-time PCR.

Concordance (n = 10)			kappa	p
FISH/RT-PCR Concordance	FISH/RT-PCR Discordance			
	FISH+/RT-PCR-	FISH-/RT-PCR+		
9 (90%)	1	0	0,615	0,035

With regard to sensitivity, RT-PCR and FISH showed very high levels of sensitivity of 89% and 100%, respectively. The specificity was excellent (100% for each) with a Youden’s index close to or equal to one **(**Table 7**)**.

**Table 7:** Sensitivity, specificity and Youden’s index for the different molecular tests.

	FISH test	RT-PCR test
Sensitivity	100%	89%
specificity	100%	100%
Youden Index	1	0,89

## 4 Discussion

STSs are a heterogeneous group of malignant and rare tumors that develop at the expense of soft tissues. Their management requires a multidisciplinary approach from the initial diagnosis, preferably in referral centers. The heterogeneity of this type of sarcoma requires multiple histological studies and sometimes a second opinion to make the diagnosis [11]. These mesenchymal tumors are characterized by specific genetic alterations that help distinguish each histological (sub)type.

Since the diagnosis of STS remains a very complicated task, recommendations and practices have been put in place for good management [12, 13]. However, observational studies [14, 15], have noted that the proper application of recommendations and practices in the management of patients with sarcoma influence survival rates. This was also shown in a recent study [16] that showed an improvement in survival rates after the use of multidisciplinary meetings. The use of the FISH test in addition to histological and immunohistochemical diagnosis, in cases of unusual pathological presentation or during a doubtful specific histological diagnosis, is one of the most indispensable recommendations. In this context, we sought to determine the impact of using this diagnostic technique on the survival time of patients with STS. The overall, metastasis-free, and recurrence-free survival rates showed no statistical significance (p = 0.15, p = 0.7, and p = 0.9, respectively) between the two groups of patients. These results show that FISH has no impact on patient survival.

Cytogenetical screening allowed the evaluation of a wide range of genetic aberrations (insertions, deletions, amplifications) in several types of cancers including STS [17]. In our study, we aimed to eliminate malignancy (lipoma/liposarcoma), confirm the histological diagnosis, and classify STS. Furthermore, in countries with limited resources, FISH is easy and cheaper and not very difficult to develop in hospital labs compared to other genetic techniques (PCR and next-generation sequencing). It is a doable and accessible technology.

Among the STSs that would benefit from an early and precise diagnosis in order to increase the survival rate, we highlight Ewing’s sarcoma. It is characterized histologically by small round cells and immunohistochemically by strong membrane staining of the CD99 glycoprotein [18, 19]. On the molecular level, it is defined by the presence of rearrangements involving the EWSR1 gene (22q12), which can be associated with the FLI1 gene located in locus 11q24 or with the ERG gene in locus 21q22 [20, 21]. In this study, rearrangement of EWSR1 was noted in just 56% of cases. Previously, Machado et al. managed to confirm Ewing's sarcoma diagnosis in 92% of cases [22]. This difference between the two studies could be explained by the number of recruited cases which can affect the statistical analysis. However, EWSR1 gene abnormalities are unspecific as they are found in many other tumors such as clear cell sarcoma, extraskeletal myxoid chondrosarcoma, round-cell myxoid liposarcoma, and myoepithelial carcinoma [23, 24, 25]. We detected one case of desmoplastic round cell sarcoma. As in others studies [26, 27], our case was characterized by an island of small round cells, typically expressing cytokeratin and desmin markers and with rearranged EWS. Extraskeletal myxoid chondrosarcoma and myoepithelial carcinoma are two entities that can present a rearrangement of EWSR1 as shown by Skalova et al. and Noguchi et al. [28] in their studies. In this cohort we detected this rearrangement in one patient with extraskeletal myxoid chondrosarcoma and another with myoepithelial carcinoma. Recently, researchers have described new entities named Ewing-like with identical morphological characteristics to Ewing’s Sarcoma, but showed different genetic rearrangements [29, 30]. We were able to classify four cases Ewing-like after a FISH test by the CIC probe. Since a differential diagnosis of Ewing's sarcoma is difficult, the EWSR1 rearrangement combined with CD99 immunostaining allowed us to give an exact classification for each patient and differentiated between the different entities. A study on a large cohort of Moroccan patients with Ewing's sarcoma showed that the combination of EWSR1 rearrangement and CD99 immunostaining is more sensitive and specific than each test alone [31]. Among all the cases that showed no rearrangement of the EWS gene, 21 cases were labeled as undifferentiated round-cell sarcomas and three cases as undifferentiated spindle cell sarcoma. To confirm the final diagnosis, it is necessary to make interpretations in the light of the clinical context and morphological/immunohistochemical analysis.

Another type of sarcoma is synovial sarcoma, which is characterized genetically by an SYT-SSX fusion gene. This gene is expected to be a molecular diagnosis marker [32, 33]. Generally, this type of sarcoma is hard to diagnose in daily practice, especially in the case of SS monophasic spindle cell and small round-cell poorly differentiated SS. These forms always require confirmation by FISH [24, 34] FISH for X:18 was performed for 37 cases diagnosed with synovial sarcoma, and the rearrangement of the SS18 gene was detected in 24 patients (65%). The other 12 cases that showed negative results were finally labeled as undifferentiated sarcomas. These results are consistent with other studies [5, 34] that have found cases diagnosed histologically as synovial sarcoma but were found negative by FISH. In recent studies, a novel SS18-SSX fusion-specific antibody (S18-SSX and SSX (C-term)) highly sensitive and specific for SS has been used in IHC and could replace molecular genetic or cytogenetic tests [35, 36].

Adipocytic tumors are the most common soft tissue neoplasia. Morphologically, they are composed of a relatively mature adipocyte proliferation. Molecular biology in combination with with histology and cytogenetics allowed us to differentiate four subcategories (well-differentiated/dedifferentiated, myxoid/round, and pleomorphic cells). These tumors are characterized by amplification of the MDM2 and CDK4 genes [37]. In daily practice, it is sometimes impossible to distinguish between a well-differentiated liposarcoma and a lipoma. The use of antibodies directed against MDM2 and CDK4 proteins remains the easiest way to confirm the diagnosis, but they are not always specific (there are subexpressions of these proteins without gene amplification) [38].

Moreover, amplification of the MDM2 gene has been reported in other malignant tumors, justifying the integration of molecular data with histological data [37]. However, the FISH test would be necessary to complement the final diagnosis of liposarcomas [38].

In our experience, we had difficulties distinguishing well-differentiated liposarcomas from benign lipomatous tumors and dedifferentiated liposarcomas from other high-grade sarcomas using only morphological criteria. This is reflected in the fact that three recurring ALT/WDLSs and three DDLS had eluded the scrutiny of our study group. To overcome this difficulty, we introduced FISH-mediated detection of MDM2 amplification in all cases of lipomatous tumors. For benign lipomatous tumors, FISH was negative for all cases, and we called them lipomas, except three cases that showed a malignant cytogenetic profile and were reclassified as WDLS. A similar study also noted two cases of ALT/WDLS that escaped histological examination [38]. Similarly, FISH for MDM2 was used to confirm the histological diagnosis of 23 WDLS, and MDM2 amplification was absent in 15 cases reclassified as undifferentiated sarcomas.

FISH for MDM2 amplification has also been used in cases with differential diagnoses of dedifferentiated liposarcoma and undifferentiated pleomorphic sarcoma. From 14 DDLSs, the diagnosis was confirmed for only seven cases while the other seven cases were reclassified as undifferentiated pleomorphic sarcomas. However, three cases diagnosed as pleomorphic sarcomas were reclassified as dedifferentiated liposarcomas after a positive MDM2 FISH. Recently, p16 IHC combined with CDK4 IHC and MDM2 amplification has been proposed as a useful diagnostic biomarker in the differential diagnosis of ALT/WDLPS and DDLPS [39].

Our work is unique in Morocco because we studied the performance of the FISH test compared to histological and immunohistochemical methods in the diagnosis of STS.

We found that FISH is highly specific (91%) but less sensitive (59%) with a high PPV of 93%. Previously, some studies with large cohorts showed that FISH is a more sensitive and specific adjunctive test to differentiate certain entities [40, 41]. This difference could be explained by the number of recruited cases that can affect the statistical analysis and the conservation of the FFPE tissue or even the duration of sample fixation that can affect the final results. However, the results demonstrate good performance for FISH in relation to the histological/immunohistochimical results with a ROC AUC value of 0.760 (95% CI: 0.69–0.82), p = 0.000. Then, we evaluated the performance of histological/immunohistochimical tests and FISH in relation to the final diagnosis. In this study, both tests were sensitive but FISH was more specific (100%) than IHC (57%). In addition, the IHC technique showed low positive and negative predictive values (66% and 91%, respectively) compared to the FISH technique (PPV = 100%, NPV = 92%).

Nonetheless, IHC has been useful to us for the selection of STS cases that may be candidates for molecular testing by FISH. Thus, this technique may not offer us the final outcome for an accurate diagnosis but it plays a crucial role in reducing the economic cost of molecular testing when caring for patients with suspected STS. Additionally, the Youden's index of FISH (0.91) was close to the value of 1, which supports the FISH technique as an accurate differential diagnostic tool.

Regarding performance, FISH showed a better performance for final diagnosis with a ROC AUC value of 0.953 (95% CI: 0.918–0.988), p = 0.000, compared to IHC with a ROC AUC value of 0.751 (95% CI: 0.683–0.820), p = 0.000. FISH has been shown in several studies to have a high performance for assessing the genomic status of different entities and presenting an accurate classification [5, 34, 42, 43].

The identification of fusion transcripts not only supports the diagnosis and classification of STS, but are particularly useful in the differential diagnosis of patients with an uncertain or unspecific morphology [44]. Certain routine techniques can be an effective aid to pathologists in the diagnosis of such neoplasms [45]. In all of the molecular advances, FISH and RT-qPCR offer valuable tools for detecting rearrangements of the SS18-SSX genes. In this context, we tried to compare FISH and RT-qPCR, which is known as a precise method. In the present study, both methods showed good concordance with a Kappa coefficient of 61.5% (p = 0.035) and they have the same specificity (100%), but FISH showed a better sensitivity than RT-qPCR (100% and 89%, respectively). It has been reported that the sensitivity and specificity of FISH in the diagnosis of synovial sarcoma are 83% and 100%, respectively [43, 46]. However, in view of one case giving a positive result by FISH, which could not be analyzed by RT-qPCR owing to a failure of amplification of cDNA, FISH is considered to complement RT-qPCR. Another study has shown that the combination of FISH and RT-PCR for detecting BCOR gene rearrangements are reliable tests and should be considered [47].

Furthermore, FISH in comparison to other genetic techniques (PCR and next-generation sequencing) is feasible and accessible technology with a rentability of tests in 95% of cases in our series (a total of 10 cases were noninterpretable due to technical reason). These results are consistent with a previous study in which 5–10% of molecular tests are noninterpretable [48]. Various pre-analytical factors can contribute to difficult-to-interpret FISH, including tissue quality, fixation methods, buffers used, and quality of paraffin material used. This confirms that the pre-analytical step is a major step that must be taken into great consideration by pathologists to obtain interpretable results.

## 5 Conclusion

The molecular classification of STS plays a crucial role in improving the quality of pathological diagnosis and, therefore, of treatment options. In daily practice, FISH is essential for adequate classification, to distinguish a benign tumor from a sarcoma, to identify a particular type of sarcoma, and to make a diagnosis for unusual clinical and histological forms.

Due to its limited resources, Morocco used to rely mainly on immunohistochemical analysis to diagnose STS, which was insufficient. Our finding supports the use of FISH test as a sensitive and specific diagnostic adjunct in cases where STS is a diagnostic consideration. Used in the right context, this valuable diagnostic tool can significantly reduce the risk of misdiagnosis and inappropriate treatment.

## Declarations

### Author contribution statement

Rhizlane El Koubaiti: Conceived and designed the experiments; Performed the experiments; Analyzed and interpreted the data; Wrote the paper.

Asmae Mazti: Analyzed and interpreted the data.

Mustapha Maaroufi, Mohammed EL Idrissi, Abdelhalim El Ibrahimi, Abdelmajid El Mrini, Touria Bouhafa and Samira El Fakir: Contributed reagents, materials, analysis tools or data.

Samia Arifi: Conceived and designed the experiments; Analyzed and interpreted the data.

Laila Chbani: Conceived and designed the experiments; Contributed reagents, materials, analysis tools or data.

### Funding statement

Laila Chbani was supported by Institute for Research on Cancer (IRC), Hassan II University Hospital of Fez Morocco (grant numbers 201881/AAP2016).

### Data availability statement

The authors do not have permission to share data.

### Declaration of interest’s statement

The authors declare no conflict of interest.

### Additional information

No additional information is available for this paper.

## References

[1] F. CDM, U. KK, et M. F (2002). Pathology and Genetics of Tumours of Soft Tissue and Bone.. http://publications.iarc.fr/Book-And-Report-Series/Who-Iarc-Classification-Of-Tumours/Pathology-And-Genetics-Of-Tumours-Of-Soft-Tissue-And-Bone-2002.

[2] B. eds WHO Classification of Tumours Editorial (2020).

[3] Abdou J., Elkabous M., M’rabti H., Errihani H. (2015). Les sarcomes des tissus mous: à propos de 33 cas. Pan Afr. Med. J..

[4] Fayette J., Blay J.Y., Ray-Coquard I. (2006). Les sarcomes des tissus mous : bonnes pratiques médicales pour une prise en charge optimale. Cancer/Radiothérapie.

[5] Asif A. (2018). Fluorescence in situ hybridization (FISH) for differential diagnosis of soft tissue sarcomas. Asian Pac. J. Cancer Prev. APJCP.

[6] Shahi F. (2017). Differentiating and categorizing of liposarcoma and synovial sarcoma neoplasms by fluorescence in situ hybridization. Iran. J. Pathol..

[7] Sugita S., Hasegawa T. (2017). Practical use and utility of fluorescence in situ hybridization in the pathological diagnosis of soft tissue and bone tumors. J. Orthop. Sci..

[8] Kaplan E.L., Meier P. (1958). Nonparametric estimation from incomplete observations. J. Am. Stat. Assoc..

[9] Mantel N. (1966). Evaluation of survival data and two new rank order statistics arising in its consideration. Cancer Chemother. Rep..

[10] Šimundić A.-M. (2009). Measures of diagnostic accuracy: basic definitions. EJIFCC.

[11] (2014). Soft tissue and visceral sarcomas: ESMO Clinical Practice Guidelines for diagnosis, treatment and follow-up. Ann. Oncol..

[12] Casali P.G., Blay J.-Y., ESMO/CONTICANET/EUROBONET Consensus Panel of experts (2010). Soft tissue sarcomas: ESMO Clinical Practice Guidelines for diagnosis, treatment and follow-up. Ann. Oncol..

[13] Bui B.-N. (2007). Standards, Options et Recommandations 2006. Prise en charge des patients adultes atteints de sarcome des tissus mous, de sarcome utérin ou de tumeur stromale gastro-intestinale. Oncologie.

[14] Wolfe C.D., Tilling K., Bourne H.M., Raju K.S. (1996). Variations in the screening history and appropriateness of management of cervical cancer in South East England. Eur. J. Cancer.

[15] Tilling K., Wolfe C.D., Raju K.S. (1998). Variations in the management and survival of women with endometrial cancer in south east England. Eur. J. Gynaecol. Oncol..

[16] Mazti A. (2021). How can a multidisciplinary approach improve prognosis of soft-tissue sarcomas of extremities?. Int. J. Surg. Oncol..

[17] Chrzanowska N.M., Kowalewski J., Lewandowska M.A. (2020). Use of fluorescence in situ hybridization (FISH) in diagnosis and tailored therapies in solid tumors. Molecules.

[18] Jo V.Y., Fletcher C.D.M. (2014). WHO classification of soft tissue tumours: an update based on the 2013 (4th) edition. Pathology.

[19] Choi E.-Y.K., Gardner J.M., Lucas D.R., McHugh J.B., Patel R.M. (2014). Ewing sarcoma. Semin. Diagn. Pathol..

[20] Ewing J. (1972). Classics in oncology. Diffuse endothelioma of bone. James Ewing. Proceedings of the New York pathological society, 1921. CA A Cancer J. Clin..

[21] Zucman J. (1992). Cloning and characterization of the Ewing’s sarcoma and peripheral neuroepithelioma t(11;22) translocation breakpoints. Genes Chromosomes Cancer.

[22] Machado I. (2009). Molecular diagnosis of Ewing sarcoma family of tumors: a comparative analysis of 560 cases with FISH and RT-PCR. Diagn. Mol. Pathol..

[23] Romeo S., Dei Tos A.P. (2010). Soft tissue tumors associated with EWSR1 translocation. Virchows Arch..

[24] Tanas M.R., Goldblum J.R. (2009). Fluorescence in situ hybridization in the diagnosis of soft tissue neoplasms: a review. Adv. Anat. Pathol..

[25] Machado I., López-Soto M.V., Rubio L., Navarro L., Llombart-Bosch A. (2015). Soft tissue myoepithelial carcinoma with rhabdoid-like features and EWSR1 rearrangement: fine needle aspiration cytology with histologic correlation. Diagn. Cytopathol..

[26] Mohamed M. (2017). Desmoplastic small round cell tumor: evaluation of reverse transcription-polymerase chain reaction and fluorescence in situ hybridization as ancillary molecular diagnostic techniques. Virchows Arch..

[27] Ladanyi M., Gerald W. (1994). Fusion of the EWS and WT1 genes in the desmoplastic small round cell tumor. Cancer Res..

[28] Noguchi H. (2010). Fluorescence in situ hybridization analysis of extraskeletal myxoid chondrosarcomas using EWSR1 and NR4A3 probes. Hum. Pathol..

[29] Mariño-Enríquez A., Fletcher C.D.M. (2014). Round cell sarcomas – biologically important refinements in subclassification. Int. J. Biochem. Cell Biol..

[30] Machado I., Navarro S., Llombart-Bosch A. (2016). Ewing sarcoma and the new emerging Ewing-like sarcomas: (CIC and BCOR-rearranged-sarcomas). A systematic review. Histol. Histopathol..

[31] Louati S., Senhaji N., Chbani L., Bennis S. (2018). EWSR1 rearrangement and CD99 expression as diagnostic biomarkers for ewing/PNET sarcomas in a Moroccan population. Dis. Markers.

[32] Norlelawati A.T. (2016). Detection of SYT-SSX mutant transcripts in formalin-fixed paraffin-embedded sarcoma tissues using one-step reverse transcriptase real-time PCR. Malays. J. Pathol..

[33] Sandberg A.A., Bridge J.A. (2002). Updates on the cytogenetics and molecular genetics of bone and soft tissue tumors. Synovial sarcoma. Cancer Genet. Cytogenet..

[34] Terry J. (2005). Fluorescence in situ hybridization for the detection of t(X;18)(p11.2;q11.2) in a synovial sarcoma tissue microarray using a breakapart-style probe. Diagn. Mol. Pathol..

[35] Baranov E. (2020). A novel SS18-SSX fusion-specific antibody for the diagnosis of synovial sarcoma. Am. J. Surg. Pathol..

[36] Zaborowski M. (2020). When used together SS18–SSX fusion-specific and SSX C-terminus immunohistochemistry are highly specific and sensitive for the diagnosis of synovial sarcoma and can replace FISH or molecular testing in most cases. Histopathology.

[37] Coindre J.-M., Pédeutour F., Aurias A. (2010). Well-differentiated and dedifferentiated liposarcomas. Virchows Arch..

[38] Kimura H. (2013). Utility of fluorescence in situ hybridization to detect MDM2 amplification in liposarcomas and their morphological mimics. Int. J. Clin. Exp. Pathol..

[39] Kammerer-Jacquet S.-F. (2017). Differential diagnosis of atypical lipomatous tumor/well-differentiated liposarcoma and dedifferentiated liposarcoma: utility of p16 in combination with MDM2 and CDK4 immunohistochemistry. Hum. Pathol..

[40] Weaver J. (2010). Can MDM2 analytical tests performed on core needle biopsy be relied upon to diagnose well-differentiated liposarcoma?. Mod. Pathol..

[41] Vargas A.C. (2019). FISH analysis of selected soft tissue tumors: diagnostic experience in a tertiary center. Asia Pac. J. Clin. Oncol..

[42] Thway K., Wang J., Swansbury J., Min T., Fisher C. (2015). Fluorescence in situ hybridization for MDM2 amplification as a routine ancillary diagnostic tool for suspected well-differentiated and dedifferentiated liposarcomas: experience at a tertiary center. Sarcoma.

[43] Vargas A.C. (2018). FISH analysis of selected soft tissue tumors: diagnostic experience in a tertiary center. Asia Pac. J. Clin. Oncol..

[44] Cerrone M. (2014). Molecular strategies for detecting chromosomal translocations in soft tissue tumors (Review). Int. J. Mol. Med..

[45] Singer S. (1999). New diagnostic modalities in soft tissue sarcoma. Semin. Surg. Oncol..

[46] Sun B. (2008). The diagnostic value of SYT-SSX detected by reverse transcriptase-polymerase chain reaction (RT-PCR) and fluorescence in situ hybridization (FISH) for synovial sarcoma: a review and prospective study of 255 cases. Cancer Sci..

[47] Li L. (2021). Detection of BCOR gene rearrangement in Ewing-like sarcoma: an important diagnostic tool. Diagn. Pathol..

[48] Neuville A. (2013). Impact of molecular analysis on the final sarcoma diagnosis: a study on 763 cases collected during a European epidemiological study. Am. J. Surg. Pathol..

